# ABA-dependent suberization and aquaporin activity in rice (*Oryza sativa* L.) root under different water potentials

**DOI:** 10.3389/fpls.2023.1219610

**Published:** 2023-09-07

**Authors:** Ga-Eun Kim, Jwakyung Sung

**Affiliations:** Deptment of Crop Science, Chungbuk National University, Cheong-ju, Republic of Korea

**Keywords:** ABA, aquaporins, drought, rice, suberization

## Abstract

Drought is one of the most stressful environments limiting crop growth and yield throughout the world. Therefore, most efforts have been made to document drought-derived genetic and physiological responses and to find better ways to improve drought tolerance. The interaction among them is unclear and/or less investigated. Therefore, the current study is to find a clue of metabolic connectivity among them in rice root experiencing different levels of drought condition. We selected 19 genes directly involved in abscisic acid (ABA) metabolism (6), suberization (6), and aquaporins (AQPs) activity (7) and analyzed the relatively quantitative gene expression using qRT-PCR from rice roots. In addition, we also analyzed proline, chlorophyll, and fatty acids and observed cross-sectional root structure (aerenchyma) and suberin lamella deposition in the endodermis. All drought conditions resulted in an obvious development of aerenchyma and two- to fourfold greater accumulation of proline. The limited water supply (−1.0 and −1.5 MPa) significantly increased gene expression (ABA metabolism, suberization, and AQPs) and developed greater layer of suberin lamella in root endodermis. In addition, the ratio of the unsaturated to the saturated fatty acids was increased, which could be considered as an adjusted cell permeability. Interestingly, these metabolic adaptations were an exception with a severe drought condition (hygroscopic coefficient, −3.1 MPa). Accordingly, we concluded that the drought-tolerant mechanism in rice roots is sophisticatedly regulated until permanent wilting point (−1.5 MPa), and ABA metabolism, suberization, and AQPs activity might be independent and/or concurrent process as a survival strategy against drought.

## 1 Introduction

Due to the climate change, drought has become one of the biggest concerns to threat growth and yield of staple crops (Shakeel et al., 2011; Zhang et al., 2018; Dietz et al., 2021). Although paddy rice requires huge amount of rainfed and/or irrigated water during the whole growing season [~3,000 L/kg of grain (Bouman et al., 2007)], rice often experiences drought due to insufficient water supply. Drought caused crop yields declines (Fahad et al., 2017), especially the loss of rice yields due to drought accounted for about 50%–60%, and it was predicted that a yield loss will be reached at 70%–80% in the future (Leng and Hall, 2019). According to the report (Maddocks et al., 2015), it is anticipated that the countries consuming rice grain as a main food will be facing to severe drought stress in 2040, and this implies the necessity to extend our knowledge on water physiology of rice root under a variety of soil water potentials.

In general, water use of plants extremely depends on the gradients between soil (highest) and plant atmosphere (lowest) (Kramer and Boyer, 1995). A severe drought results in the reverse of water flow between soil and plant and, thus, plants meet with a temporary (−1.0 MPa) and/or permanent (−1.5 MPa) wilting points (Richards and Weaver, 1943). Plants cannot utilize water in soil with ≤ −1.5 MPa including hygroscopic water (−3.1MPa). Plants are autotrophs that synthesize their own energy and use it for themselves. Plant’s energy produced by photosynthesis cannot exceed a certain level (Zhu et al., 2008), and energy synthesis efficiency is negatively affected when plants exposed to drought stress (Chaves et al., 2002). Therefore, plants actively and precisely utilize their own energy to not only growth and development but also stress-derived resistant mechanism. However, severe stress results in irreversible damage and death to plants (Mosa et al., 2017).

Rice responds to drought by changing their phenotype (Muthurajan et al., 2018) or gene expression levels (from growth to stress tolerance). The phytohormone abscisic acid (ABA) plays an important role in regulating rice responses to biotic and abiotic stresses, and osmotic stress induces a marked accumulation in root (Shi et al., 2015). Osmotic stress-derived ABA quickly induces stomatal closure and reduces shoot growth (Cardoso et al., 2020). Suberin is defined as a glycerol-based aliphatic polyester complex, and suberization that is a physical process of suberin lamellae deposition is a layer formed between cell wall and plasma membrane after development of the casparian strip (CS) (Enstone et al., 2002). Suberin lamellae regulate the movement of water and nutrients by blocking all pathways for water flow in the roots except the symplastic pathway (Franke and Schreiber, 2007; Barberon et al., 2016). The accumulation of suberin lamellae increases in drought-exposed roots, which is known to be a tolerance mechanism to drought in rice and barley (Henry et al., 2012; Kreszies et al., 2019). As recently discovered, ABA induces the activation of suberization in roots (Wang et al., 2020). However, ABA-derived root suberization mechanism under various water potentials provides limited knowledge.

Furthermore, ABA is known to be involved in the regulation of aquaporins (AQPs) in drought (Ding et al., 2016). AQPs, water channel proteins, are the membrane proteins that transport water molecules in all organisms (Zardoya and Villalba, 2001; King et al., 2004) and are essential for maintaining a homeostasis under a perturbation of water potentials at cellular level (Shivaraj et al., 2021). AQPs, a crucial key of root water transport, have been studied with histological localization (Sakurai et al., 2008) and gene expression (Nguyen et al., 2013) in rice. Interestingly, the expression of AQPs genes was greatly dependent upon along the root axis in relation with barriers (CS and suberin lamellae) (Wang et al., 2019). Nevertheless, the physiological interaction between suberin lamellae deposition and aquaporin’s function is unclear, especially under limited water supply. Based on previous reports (Mahdieh et al., 2008; Šurbanovski et al., 2013), the expression of aquaporin genes in drought is not only greatly differed from plant species and stress conditions such as a strength and period but also is closely involved in root hydraulic conductivity.

Despite a lot of valuable reports to water stress-derived physiological responses in plant root, there is still limited information, which is comprehensively dealing with the relationship between ABA-responsive suberization and aquaporins. We hypothesize that suberization and aquaporin could be precisely regulated and/or closely connected with ABA-signaling mechanism under drought, because both processes are directly employed in water transportation across cell membrane. To extend our knowledge on the role of endogenous ABA about both suberization and aquaporin activity for perception of the water gradient, we focused on the transcriptional changes in ABA to suberization-associated genes and aquaporins from rice roots. These transcriptional data were also supported by assaying the relative levels of fatty acids, considered as intermediates for suberization.

## 2 Materials and methods

### 2.1 Plant material and water deficit through PEG-6000

Rice (*Oryza sativa* L. cv. “Saechucheongbyeo”) seeds were imbibed and sterilized with 5 L of distilled water containing 2.5 mL of a seed sterilizer (Kimaen, Farm Hannong, Korea) for 24h at room temperature and germinated in an incubator (darkness, 28°C) for 5 days. The uniformly grown seedlings (10 plants per treatment) were carefully transplanted into a hydroponic system containing 1/2 strength (0.5×) of Hoagland nutrient solution (Hoagland and Arnon, 1950) and grown until reaching at fifth leaf stage (3 weeks) with replacing a liquid media every week. The growth condition of a growth chamber (VS-91G09M-2600, VISION SCIENTIFIC Co. Ltd., Korea) was fixed with a 14/10h photoperiod, 60% of relative humidity (RH) and 27/27°C (day/night) temperature.

To develop the gradient of drought stress, water potential (Ψs) was firstly adjusted with 0 (0% of PEG-6000), −1.0 (8%), −1.5 (10%), and −3.1 (20%) MPa using polyethylene glycol (PEG)-6000 with 1/2 strength (0.5×) of Hoagland nutrient solution. Rice seedlings were exposed for 7 days with different strength of drought, and the shoot and root were carefully harvested and stored at −80°C until further analysis (six to eight plants per treatment).

### 2.2 RWC, proline, and chlorophyll analysis

The relative water content (RWC) was calculated as the following formula. RWC (%) = (fresh weight − dry weight)/(turgid weight − dry weight) × 100. The fresh weight of fully developed leaves of each plant was measured, and the turgid weight was measured from leaf disc (1 × 1 cm) immersed in distilled water at 4°C for 3h to minimize respiratory loss. The dry weight was determined after dryness for 48h at 80°C.

The proline was analyzed with a previous method (Carillo and Gibon, 2011). The fresh samples (0.5 mg) from rice shoots and roots were mixed with 1 mL 40% (w/v) ethanol at 4°C for 24h, centrifuged at 14000*g* for 5 min, and the supernatant was extracted. The supernatant (500 µL) was immediately incorporated into a reaction solution containing 3% sulfosalicylic acid, 1 mL of glacial acetic acid and 1 mL of acidic ninhydrin, incubated at 96°C for 1h, and the reaction was terminated on ice. Toluene (1 mL) was added to the reaction mixture, and the supernatant was used as an analytical sample for measuring proline using a spectrophotometer (520 nm) (UV-1900i, Shimadzu, Japan) after vortexing for 20 s and standing for 5 min. The L-proline was used as a standard (Sigma, MO, USA). The proline content was calculated using a method by Augusto et al. (Ramírez-Godoy et al., 2018): Proline content (µg per g of fresh sample) = [(µg Proline/mL × mL Toluene)/115.5 µg/µmol]/[(g sample)/5].

Chlorophyll contents were analyzed according to the method of Arnon (Arnon, 1949). Fresh leaves (0.5 g) were immersed in 25 mL of 80% (w/v) acetone. Mixtures were vortexed and incubated for 24h at darkness. The absorbance of supernatants was measured at 645 nm and 663 nm using spectrophotometer (UV-1900i, Shimadzu, Japan). The chlorophyll content was calculated as following formula:


Absorbance was Chlorophyll a (mg per g of fresh sample) = (12.7 × D663 − 2.69 × D645) × DF

Chlorophyll b (mg per g of fresh sample) = (22.9 × D645 − 4.68 × D663) × DF

Chlorophyll total (mg per g of fresh sample) = (20.2 × D645 + 8.02 × D663) × DF

DF = Dilution factor = (V/1000) × W

V = final volume of chlorophyll extract in 80% acetone

W = fresh weight of tissue extracted

### 2.3 Microscopic observation of root suberization

Suberin lamellae were microscopically observed from the crown roots of rice seedlings exposed for 7 days under different water potentials (0, −1.0, −1.5, and −3.1 MPa). Root was divided into three developmental zones, division to elongation (0 mm–30 mm from root apex), elongation to maturation (30 mm–50 mm), and maturation (50 mm–100 mm), and quickly fixed with methanol. Suberin lamellae-specific staining are as follows: (1) first staining for 1h in methanol including 0.01% of fluorol yellow 088 (w/v) in darkness with gently agitation, (2) rinsing with methanol and second staining in methanol including 0.5% aniline blue (w/v) for 1h at room temperature in darkness, and (3) rinsing with methanol and mounting on water. Specimens were observed as a green fluorescence excited by ultraviolet (UV) light using a florescence microscope (×10, Eclipse Ti, Nikon, Tokyo, Japan).

### 2.4 Extraction and analysis of fatty acids

The extraction of fatty acids was carried out according to a previous procedure (Park et al., 2021). Briefly, root sample (0.1 g, powdered) was mixed with 0.7 mL of chloroform:methanol solution (2:1, v/v), 0.7 mL of 0.58% sodium chloride, and 0.1 mL of pentadecanoic acid in chloroform as IS (1 mg/mL). The sample was vortexed for 30 s, and centrifuged at 15,000*g* at 4°C for 5 min. The under layer was transferred into an e-tube (2 mL) and dried using a vacuum concentrator. After dryness, the extract was incubated with 0.1 mL of toluene, 0.18 mL of methanol, and 0.02 mL of 5 M sodium hydroxide at 85°C for 5 min. After cooling for 3 min, the mixture was catalyzed by mixing 0.3 mL of boron trifluoride at 85°C for 5 min, and 0.8 mL of pentane and 0.4 mL of distilled water were blended with the samples. The mixture was centrifuged for 15 min at 350*g* at 4°C, and the supernatant was transferred to an e-tube (2 mL). The concentrated sample was resolved in 0.1 mL of hexane. The methylated sample (1 μL) was directly injected into the gas chromatography-mass spectrometry (GC-MS, Shimadzu, Japan). For separation of medium and long chain fatty acids, a Rtx-5MS column (30 m × 0.25 mm id, film thickness 0.25 μm, Restek, Bellefonte, PA, USA) was used. The temperature was initiated with 40°C for 2 min, increased to 320°C at a rate of 1°C/min, and finally maintained at 320°C for 6 min. A flow rate of helium as a carrier gas was 1.42 mL/min, and full scan mode (m/z 45–500) was used. The GC-MS software (version 4.11; Shimadzu, Kyoto, Japan) was used. The results were adjusted with their retention times and mass spectra with reference to standard compounds and the in-house library. Quantitative analysis was conducted using the ratio of the analyte peak area to the IS peak area.

### 2.5 Quantitative real-time PCR

Total RNA was extracted from the roots of rice seedlings at 7 days after the initiation of drought, 0, −1.0, −1.5 and −3.1 MPa, respectively, using TRIzol reagent (Invitrogen, Carlsbad, CA) according to the manufacturer’s instructions. The purity and concentration of the extracted RNA were estimated using NanoDrop (Thermo Fisher Scientific, Madison, WI, USA) and checked on a 1.2% agarose gel. Total RNA (1 μg) and the RT PreMix Kit with Oligo (dT) primers were used to synthesize first-strand cDNA using following PCR condition: 60 min at 45°C to cDNA synthesis and 5 min at 95°C to Rtase inactivation step.

Quantitative real-time PCR was performed by using a Real-Time PCR machine (CFX Opus 96, Bio-Rad, Hercules, CA, USA) with technical triplicates according to the manufacturer’s instructions. The reaction mixture consisted of 1 μL of cDNA template, 2 μL each of 10 mM forward and reverse primer (**Supplementary Table S1**) and 5 μL SYBR Green Q Master mix (Labopass, Cosmo Genetech, Seoul, South Korea). The PCR conditions consisted of pre-denaturation step at 95°C for 5 min, followed by 50 cycles of denaturation 95°C for 10 s, annealing temperature of each primer (**Supplementary Table S1**) for 30 s and elongation (72°C, 20 s). This step was followed by a melting curve, ranging from 65°C to 95°C at a heating rate of 0.5°C/s. A quantification method (2^–ΔΔCt^) was used (Livak and Schmittgen, 2001) and the variation in expression was estimated using triplicate for each cDNA sample. The rice actin gene was used as a reference in the qRT-PCR. Primer sequences used for qRT-PCR were designed by Primer 3 software (Rozen and Skaletsky, 2000).

### 2.6 Statistical analysis

Statistical analysis was conducted using R statistical software version 4.0.3 and R studio version 1.3.1093. Data are presented as mean and standard deviation. Data were compared using one-way ANOVA and Tukey’s honest significant difference (HSD) test. For the data from qRT-PCR and fatty acid analysis, statistics were performed using SAS software version 9.4 (SAS Institute, Cary, NC, USA), and the significance was tested with Fisher’s least significant difference (LSD) test at the 5% level of probability.

## 3 Results

### 3.1 Rice growth and root aerenchyma development under different water potentials

**Figure 1:**
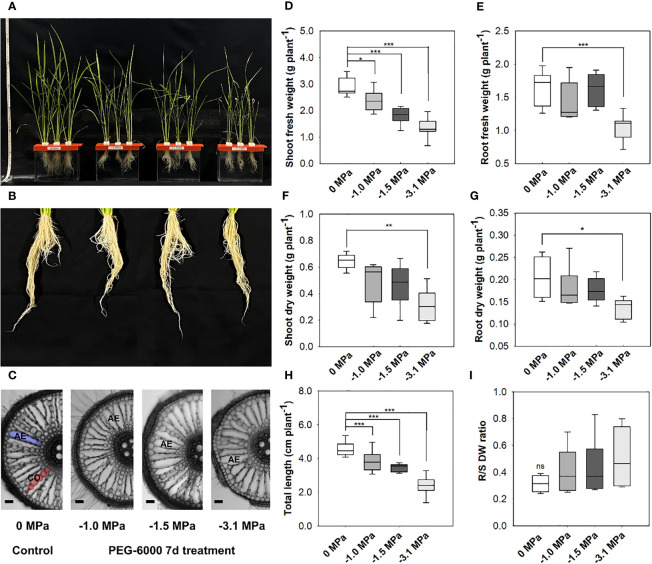
Rice growth and root aerenchyma modification under different water potentials, 0, −1.0, −1.5, and −3.1 MPa, for 7 days **(A, B)**. PEG-6000 was used to develop the gradient of water potential (Ψs), −1.0 MPa (8% of PEG-6000), −1.5 (10%), and −3.1 (20%), respectively. A growth media with different water potentials commonly includes 1/2 strength (0.5 ×) of Hoagland nutrient solution, and was replaced every week. Aerenchyma development of rice root. The microscopic observation was a 50 mm from the root apex (Bar = 100 µm) **(C)**. AE = Aerenchyma. Biomass [fresh weight, **(D, E)**; dry weight, **(F, G)**], total plant length **(H)** and ratio of root to shoot (dry weight) **(I)**. Statistical significance by Tukey test: ns, no significance; *p < 0.05; **p < 0.01; ***p < 0.001 (*n* = 5).

In the current study, we employed four levels of water potential (PEG-6000-derived), 0, −1.0, −1.5, and −3.1 MPa, respectively. The growth and wilting (dehydration) of rice seedlings were significantly affected by the strength of water stress (**Figure 1**). A visible damage by water stress was marked in the shoot rather than the root (**Figures 1A, B** **)**. Aerenchyma formation was shown at 50-mm distance from the crown root tip (**Figure 1C**). The roots at −1.0, −1.5, and −3.1 MPa were likely to develop more aerenchyma spaces than well-watering condition (0 MPa), although the aerenchyma did not represent the difference between the levels of water stress. The growth (fresh weight based) of rice shoot was significantly decreased at drought condition, whereas root was significantly decreased only at -3.1MPa (**Figures 1D, E** **)**. The limited growth (dry weight-based) of rice seedlings was significant at −3.1 MPa (**Figures 1F, G** **)**, which indicated 50% of growth reduction. Plant total height was remarkably affected in all treatment groups, 7% reduction at −1.0 and −1.5 MPa and 20% at −3.1 MPa (**Figure 1H**). The R/S ratio was a trend of a decrease under water stress although there was no significant difference between water potentials (**Figures 1A–I**).

### 3.2 Physiological indicators of rice seedlings under different water potentials

**Figure 2:**
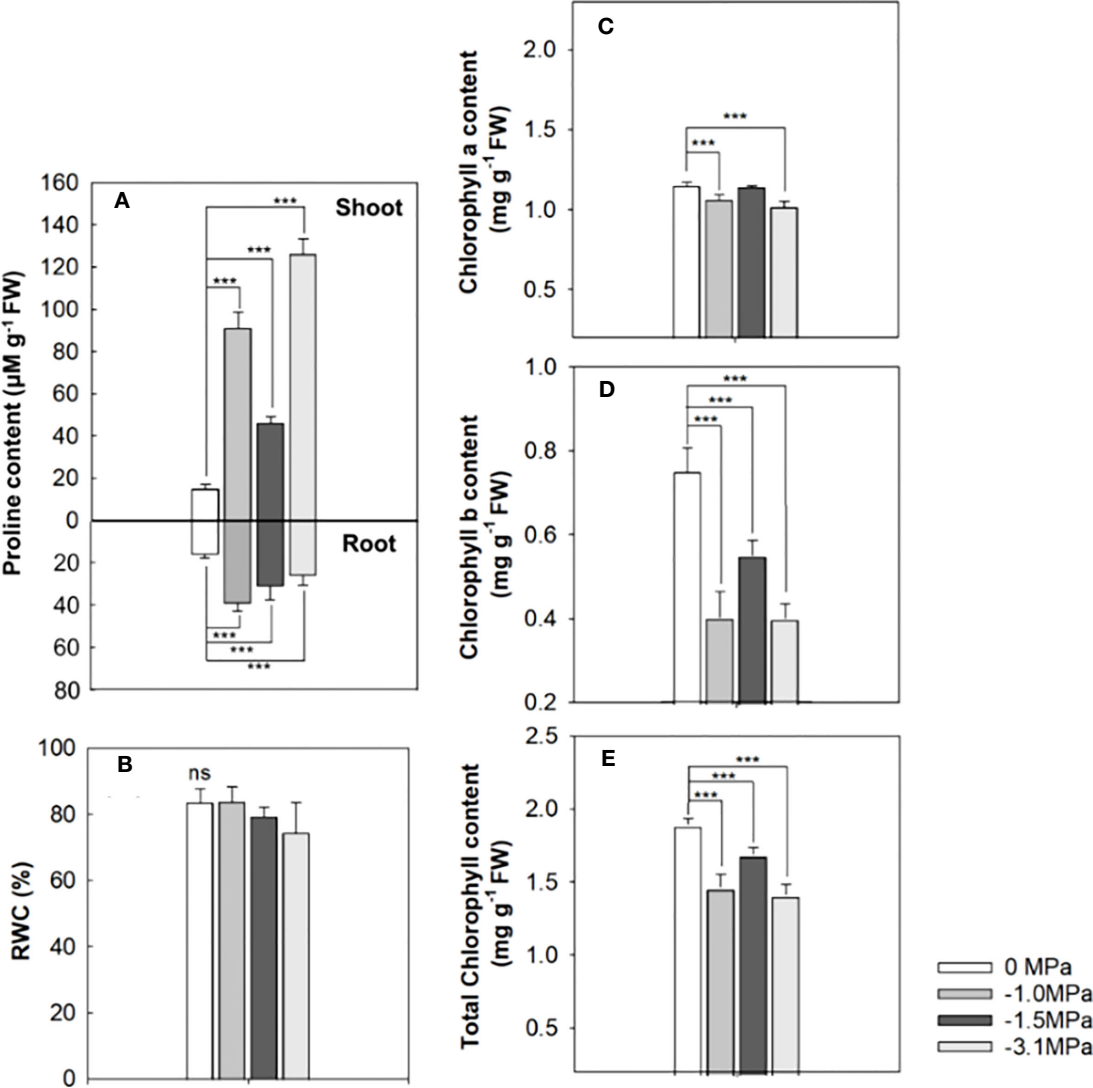
Proline **(A)**, relative water content (RWC, **(B)**), chlorophyll a **(C)**, chlorophyll b **(D)**, and total chlorophyll **(E)** of the rice seedlings treated with different water potentials, 0, −1.0, −1.5, and −3.1 MPa for 7 days. Each bar represents average ± standard deviation (*n* = 5). Statistical significance by Tukey test: ns, no significance; ***p < 0.001.

Proline, an osmoprotectant, was markedly accumulated in both shoot and root by water stresses, and the highest level showed at −3.1 MPa in the shoot and −1.0 MPa in the root (**Figure 2A**). The level of proline was 45–120 µmol g^−1^ (fresh weight, FW) and 30–40 µmol g^−1^ in water stressed shoots and roots, respectively. The accumulation of proline in both organs did not depend on the drought strength. The RWC was not differed from all water potentials (**Figure 2B**). Chlorophyll contents were significantly decreased by water stress (**Figures 2C–E**), and the reduction was remarkable in chlorophyll b, indicating 50%–60% of decrease in all water potentials. Chlorophyll a content by water stress was 1.0–1.1 mg g^−1^ (FW), whereas chlorophyll b showed a range of 0.3–0.5 mg g^−1^.

### 3.3 ABA biosynthesis and signaling in rice roots under different water potentials

**Figure 3:**
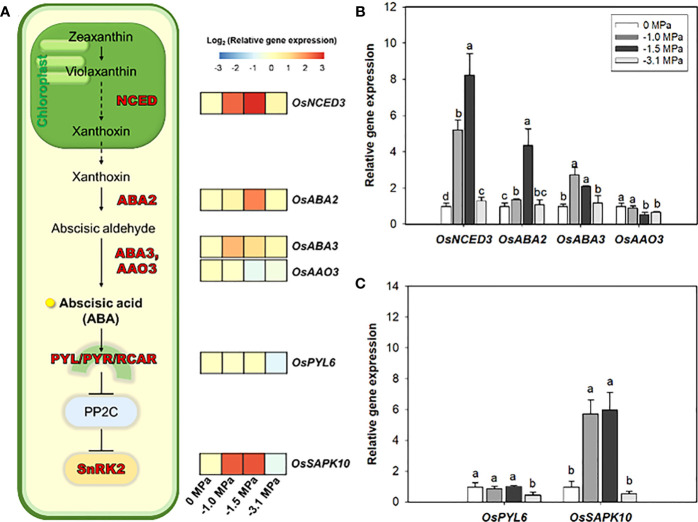
Heatmap of relative gene expression of ABA biosynthesis and signaling in rice roots treated with different water potentials, 0, −1.0, −1.5, and −3.1 MPa, for 7 days **(A)**. Relative expression levels (log_2_ scale) of ABA biosynthesis **(B)** and signaling **(C)** genes by qRT-PCR (*n* = 3). Letters above bars represent significant difference (*p* < 0.05) by Fisher’s least significant difference (LSD) test. NCED, 9-cis-epoxycarotenoid dioxygenase; ABA2, A short-chain dehydrogenase/reductase (SDR); ABA3, Molybdenum Cofactor Sulfurase; AAO3, abscisic aldehyde oxidase 3; PYL, pyrabactin resistance (PYR)1-LIKE; PP2C, Type 2C protein phosphatase; SAPK, Stress/ABA-activated protein kinases.

Based on current knowledge, we analyzed the transcriptional variations of some key genes, which are directly involved in ABA biosynthesis and signaling, from rice roots under different water potentials (**Figure 3**). Both water potentials, −1.0 and −1.5 MPa, resulted in the significant upregulation of *OsNCED3* (ABA biosynthesis), which indicated 5.0- and 8.0-fold higher water potential, respectively. Moreover, *OsABA2* and *OsABA3* also showed noticeable increase, 1.3- to 4.0-fold. Meanwhile, *OsAAO3* remained unchanged or slightly decreased compared with the control (0 MPa). The *OsSAPK10* (encoding SnRK2), ABA-signaling gene, was significantly promoted (6.0- to 8.0-fold, log_2_ scale) by limited water supply (−1.0 and −1.5 MPa).

### 3.4 Suberin-lamellae deposition, suberization-related gene expression, and fatty acid composition in rice roots under different water potentials

**Figure 4:**
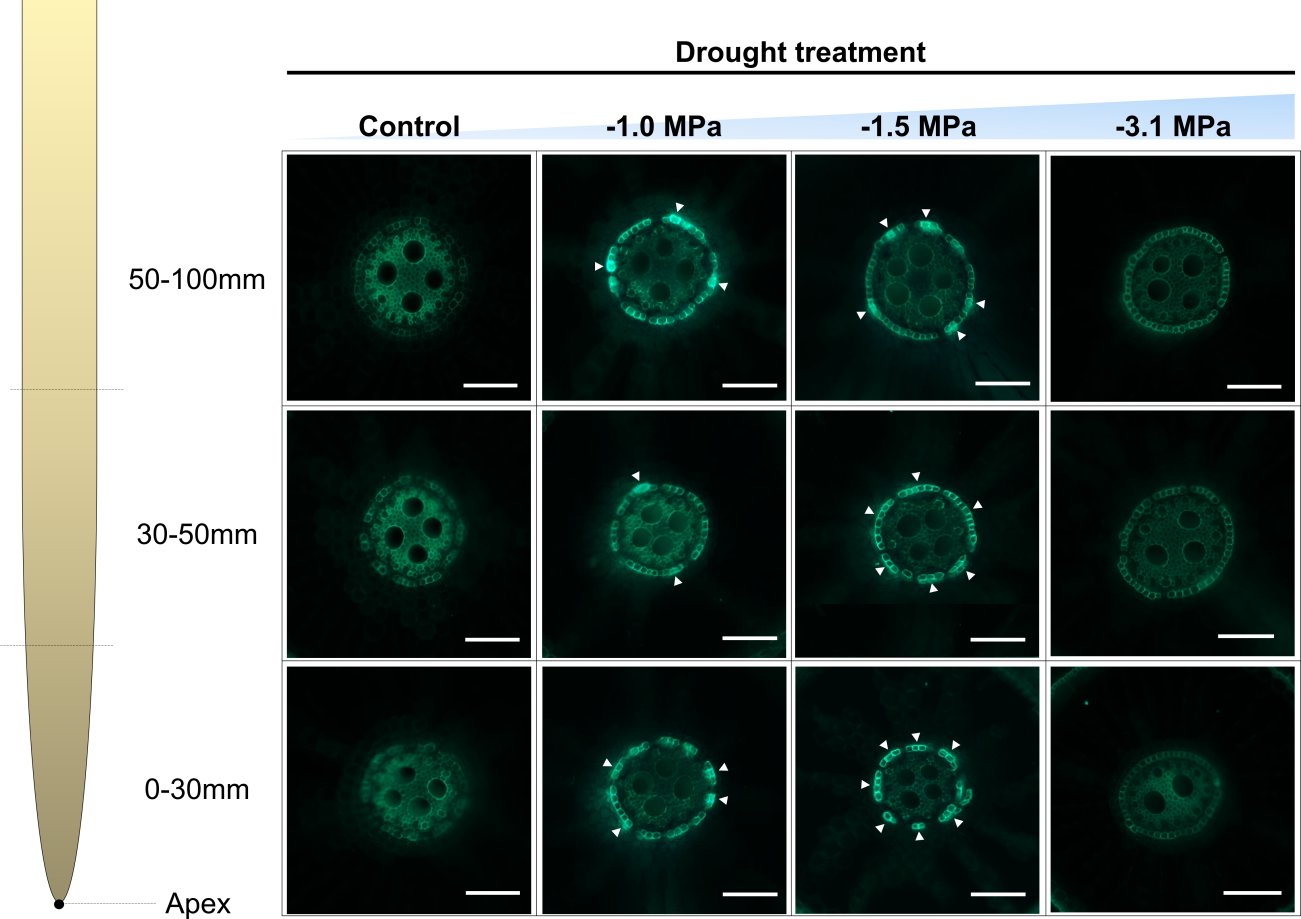
Development of suberin lamellae in rice roots treated with different water potentials, 0, −1.0, −1.5, and −3.1 MPa for 7 days. Freehand cross sections, 0 mm–30 mm, 30 mm–50 mm, 50 mm–70 mm from the root tip, were made from 1-month-old rice roots grown in hydroponics, were stained with flourol yellow 088 and aniline blue, and observed with UV illumination. Arrowheads indicate suberin lamellae. Bars = 0.1 mm.

**Figure 5:**
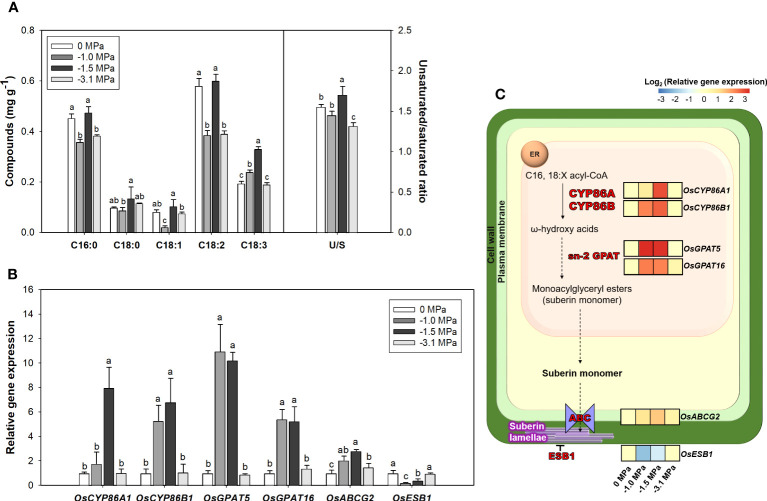
**(A)** Fatty acid contents (mg g^-1^) in rice roots treated with different water potentials, 0, −1.0, −1.5, and −3.1 MPa for 7 days. U/S (ratio of unsaturated to saturated fatty acids) = (%, total unsaturated fatty acids)/(%, total saturated fatty acids). Graph **(B)** and heatmap **(C)** of relative expression of suberization genes (qRT-PCR) converted to log_2_ scale. Each bar represents the average of three biological replicates ± standard deviation (*n* = 3). Letters above bars represent significant difference (*p* < 0.05) by Fisher’s least significant difference (LSD) test. CYP86A9, the cytochrome P450 fatty acid ω-hydroxylase; CYP86B1, cytochrome P450; GPATs, sn-2 glycerol-3- phosphate acyltransferases; ABCG, ATP binding cassette (ABC) transporter; ESB1, enhanced suberin 1.

Based on enhanced expression of ABA-biosynthetic and -signaling genes (**Figure 3**), we tried to understand the inter-communication mechanism between ABA and suberization. Suberin lamella, which is a product by accumulation of suberin in the root endodermis, is known as a tolerant mechanism against osmotic stress (Kreszies et al., 2018). The suberization in the root endodermis was markedly developed by increasing water limitations (−1.0 and −1.5 MPa) compared with well watering (0 MPa) (**Figure 4**). The −1.0 and −1.5 MPa of water potential showed the thicker layer (green fluorescence) than 0 MPa. In addition, the strength of fluorescence in the most negative water potential (−3.1 MPa) was similar to 0 MPa, which implied less suberization. Therefore, due to that −3.1 MPa did not show significant variations, which were considered as a senescence or death, our observations are mainly described and interpreted with physiological changes in −1.0 and −1.5 MPa. The selected fatty acids (**Figure 5A**) and suberization-associated genes (**Figures 5B, C**) in different water potentials experiencing rice root were analyzed to validate suberization by microscopic observation. The composition of fatty acids was greatly perturbed by decreasing water potentials (**Figure 5A**). The fatty acids (16:0, 18:1, and 18:2) in −1.0 MPa significantly decreased, whereas those in −1.5 MPa was not perturbed. On the other hand, α-linolenic acid (18:3), the highest unsaturated form, was obviously accumulated in both −1.0 and −1.5 MPa compared with the control (0 MPa). Therefore, the ratio of the unsaturated to saturated forms was markedly enhanced by limited water supply conditions. The expression of suberization-involved genes showed a similar tendency with microscopic observation and fatty acid composition (**Figures 5B, C**). The key genes, *OsCYP86A1*, *OsCYP86B1*, *OsGPAT5*, and *OsGPAT16*, to produce suberin monomer from fatty acids were significantly upregulated (2.0- to 10.0-fold-higher, log_2_ scale) in −1.0 and −1.5 MPa of water potential, whereas those in −3.1 MPa remained unchanged. An *OsABCG2*, transporting suberin monomer into cell wall layer, was also markedly promoted in both water potentials. By contrast, *OsESB1*, restricting the suberin lamella deposition, was significantly downregulated in both water potentials.

### 3.5 Aquaporins activity in rice roots under different water potentials

**Figure 6:**
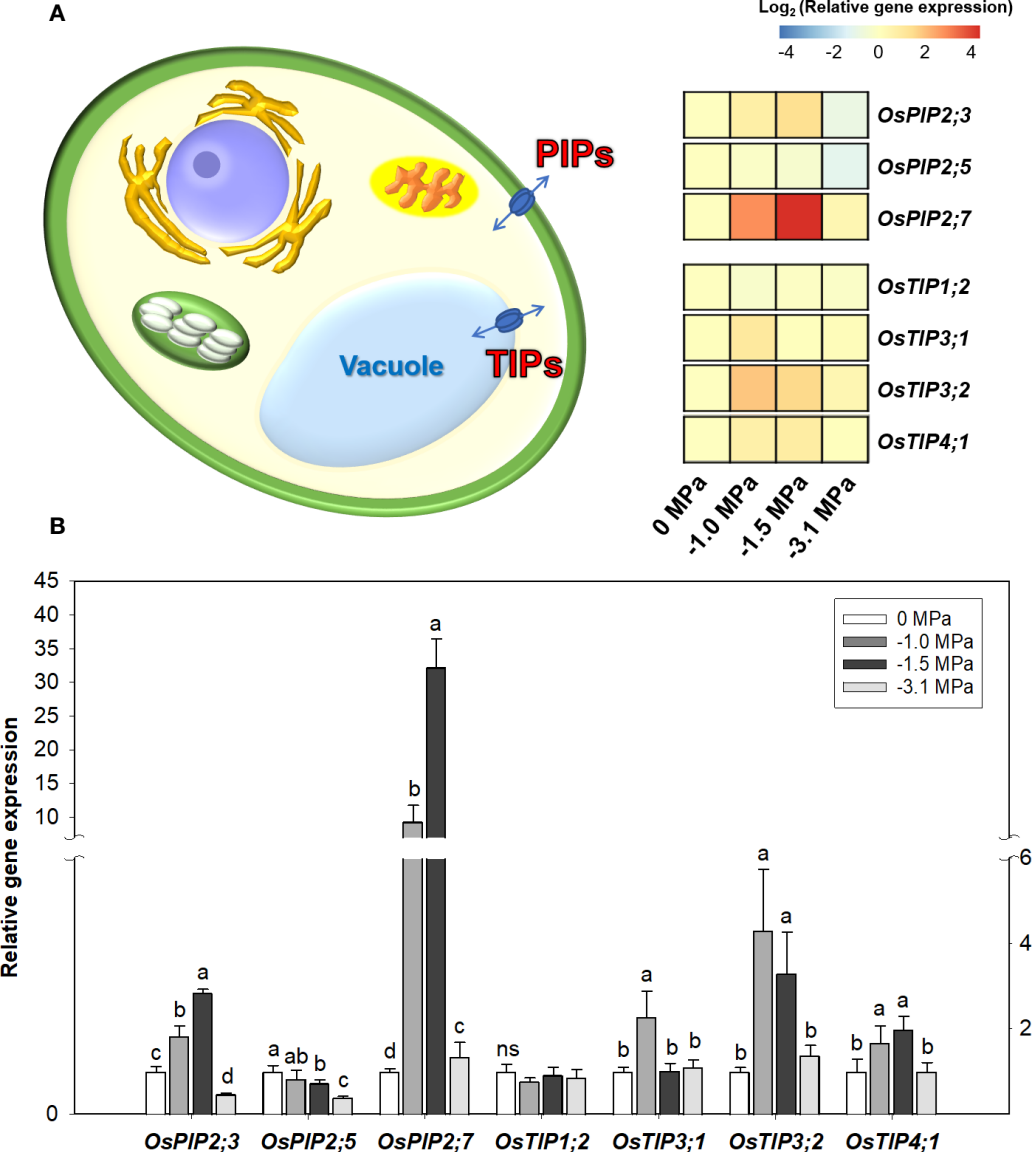
Heatmap of relative gene expression of aquaporins in rice roots treated with different water potentials, 0, -1.0, -1.5 and -3.1 MPa, for 7 days **(A)**. Relative expression levels (log2 scale) of selected aquaporin genes **(B)** by qRT-PCR (n=3). Letters above bars represent significant difference (p < 0.05) by Fisher’s least significant difference (LSD) test. PIPs, plasma membrane-intrinsic proteins; TIPs, tonoplast-intrinsic proteins.

The experience of drought during plant growth noticeably perturbs not only ABA-derived metabolisms including suberization but also AQPs response. However, there is still limited information and/or unclear information on metabolic cooperation between ABA and AQPs. The expression of selected seven genes encoding plasma membrane-intrinsic proteins (3, PIPs) and tonoplast-intrinsic proteins (4, TIPs) from rice roots was compared between different water potentials (**Figure 6**). The transcriptional changes were the very similar to those in ABA (**Figure 3**) and suberization (**Figure 5**). Firstly, the PIPs-encoding genes, *OsPIP2;3* and *OsPIP2;7*, significantly increased, and an abundance showed 2.0- to 3.0-fold greater in *OsPIP2;3* and 10.0- to 32.0-fold higher in OsPIP2;7. Many studies reported the enhanced AQPs activity under varying drought conditions. Additionally, three TIPs-encoding genes, *OsTIP3;1*, *OsTIP3;2*, and *OsTIP4;1*, also markedly increased in both drought conditions (−1.0 and −1.5 MPa), which is being ranged from two- to fourfold higher (log_2_ scale).

**Figure 7:**
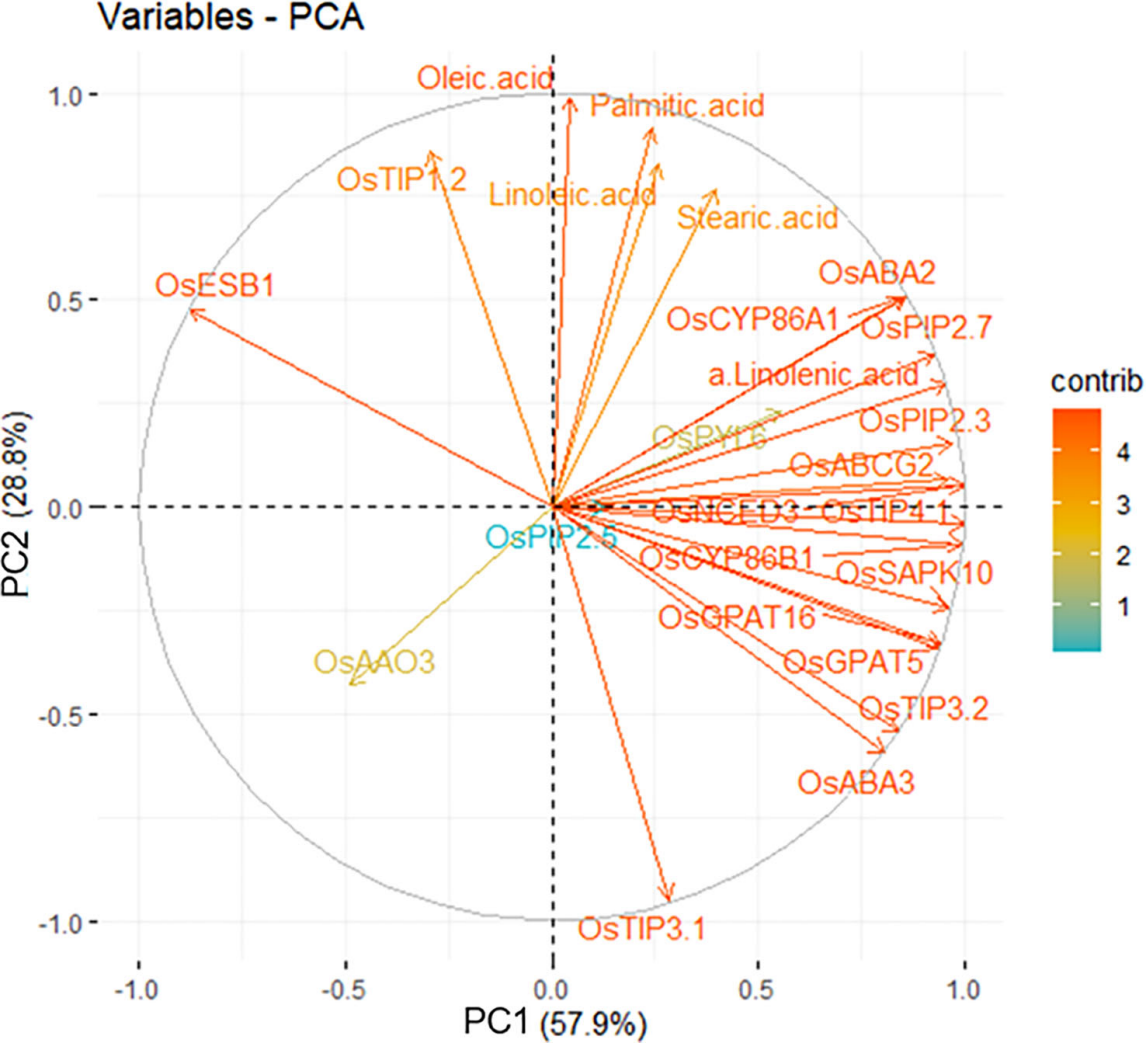
Interrelationship between water stress-derived physiological variables by principal components analysis (PCA). The variables indicate genes (ABA, suberization and AQPs) and fatty acids. The contribution of the variables is indicated from red (high) to blue (low) arrows.

In order to validate our observation, we employed the PCA with variables, and unsaturated fatty acids and all genes except for *OsESB1* were loaded on the same group by PC1, which is covering 57.9% of a validation (**Figure 7**). Therefore, the current results imply that ABA biosynthesis and signaling, suberization, and AQPs activity are directly and/or indirectly associated under the limited water condition and precisely regulated by each other.

## 4 Discussion

Water potentials of −1.0 and −1.5 MPa are in the range that plants are available to adjust from soil (Michel and Kaufmann, 1973), whereas −3.1 MPa leads to the permanent wilting to plants that result in irreversible damage and death to plants (Mosa et al., 2017). In context of water availability of plants, we primarily focused on elaborating the physiological responses, which were derived from −1.0 to −1.5 MPa different from −3.1MPa.

### 4.1 Growth and physiological responses under drought condition

In our study, the development of aerenchyma was observed in all drought treatments but could not be quantified (**Figure 1C**). Indeed, drought strongly developed aerenchyma development in roots via a degradation of cortex (Singh et al., 2013; Fonta et al., 2022) and, in context of previous observations, rice roots under drought are likely to operate the drought-tolerant strategy via precise regulation of both apoplastic and symplastic water flux. The height and dry weight of plants were largely influenced (decrease) under the limited water supply (Nasrin et al., 2020). Our study showed that the total plant length and dry weight was significantly reduced in root and shoot of the −3.1MPa (**Figures 1F–H**). However, there was no significant difference in dry weight at −1.0 and −1.5 MPa (**Figures 1F, G**).

Proline and chlorophyll are physiological indicators to determine whether plants are experiencing water stress such as drought (Shi et al., 2017; Gu et al., 2019). Proline, an osmo-protectant, is accumulated (Yoshiba et al., 1997), whereas chlorophyll is decreased in drought stress (Li et al., 2019). The positive relationship between increasing drought conditions and proline accumulation was extensively demonstrated (Sun et al., 2011), and proline revealed greater abundance in shoots compared with roots, even though it was accumulated in both tissues (Pandey et al., 2004). Our study has shown that proline more accumulated in shoot than root, and chlorophyll was decreased in drought conditions (**Figure 2**). Recently, it was suggested that proline plays a role as an energy adjuster on stress responses including drought, and ABA is closely involved in this mechanism (Alvarez et al., 2022). Furthermore, water scarcity resulted in a decrease in chlorophyll content as a result of chlorophyll degradation due to the destruction of photosynthesis apparatus by ROS generation. In particular, chlorophyll b is more sensitive to stress condition because of the conversion of chlorophyll b to chlorophyll a under drought, and thus a decrease in chlorophyll b is prominent response to drought (Rüdiger, 2002). Our observations showed the same pattern in proline and chlorophyll contents, and, therefore, it is evident that the perturbation of those biochemicals was typical physiological response to the limited water supply.

### 4.2 ABA biosynthesis and signaling mechanisms in the root under drought condition

ABA, which is well-known as a key plant hormone to drought, is directly involved in an activation of a variety of transcription factors responsible for stress condition (Zhang et al., 2006; Singh and Laxmi, 2015). This is because most of the transcription factors activated in drought are responsive to ABA (Singh and Laxmi, 2015). In addition, ABA also triggers the metabolism for root suberization via a cascade of PYL/PYR/RCAR - PP2C - SnRK2 (Wang et al., 2020; Wan et al., 2021; Shiono et al., 2022). This has led to studies of increasing drought tolerance using ABA biosynthesis and signaling enzymes and has been conducted until recently (Lu et al., 2009; Verma et al., 2019). qRT-PCR was performed by selecting six enzyme genes involved in ABA biosynthesis and signaling process (**Figure 3**). Four genes involved in ABA precursor- and ABA-biosynthesis and two ABA-signaling genes *OsPYL6* (Yadav et al., 2020) and *OsSAPK10* (Wang et al., 2017) known to be expressed in roots were analyzed. In our study, *OsPYL6* was significantly down-regulated in −3.1MPa (**Figure 3**). This suggest that drought tolerance mechanisms involving ABA are not fully activated in −3.1MPa (permanent wilting point). Whereas, in −1.0 and −1.5 MPa, the expression of ABA biosynthesis genes (*OsNCED3*, *OsABA2*, and *OsABA3*) and *OsSAPK10* were upregulated. Among them, *OsNCED3* and *OsSAPK10* were particularly highly upregulated under mild stress. These results are the same conclusion as the previous study of *OsNCED3* (Huang et al., 2018), and it can give more strength to the claim that *OsNCED3* is responsible for ABA accumulation and indirectly regulates ABA-dependent genes during water stress. It was demonstrated that *OsNCED3* and *OsSAPK10* indirectly regulate ABA-dependent cascading pathway in rice roots experiencing drought stress.

### 4.3 Suberization in the root under drought condition

Abiotic stresses including drought affect the composition of membrane lipids in plants, and thus an abundance in unsaturated forms increases (Upchurch, 2008). Various levels of water limitation resulted in an accumulation of the unsaturated fatty acids in the roots of grapevine (Zhang et al., 2020) and almond (Elloumi et al., 2014). The enhanced ratio of the unsaturated to saturated fatty acid is clearly observed as a drought-tolerant mechanism in many plants (Moradi et al., 2017; Jin et al., 2022) including rice (Lenka et al., 2011) and maize (Zi et al., 2022). Furthermore, ABA-mediated metabolism predominantly promoted the substances of suberization enhanced the activity of C18:3-ACP (Wang et al., 2022). By contrast, the extreme drought condition resulted in the reduced membrane fluidity causing less function of cell membrane (Rawat et al., 2021).

In this study, genomic and anatomical analyses were performed to compare the development of suberin lamellae according to drought level (**Figures 4**, **5**). The genes used for qRT-PCR were selected with reference to the following. Enzymes required for biosynthesis of aliphatic suberin precursors include fatty acyl ω-oxidases of CYP family and acyltransferases of the GPAT family (Li-Beisson et al., 2013). AtCYP86A1 and AtCYP86B1 are required for biosynthesis of C16-C18 and C22-C24 ω-oxygenated fatty acid (Li et al., 2007; Molina et al., 2009) and have high expression correlation with AtGPAT5 (Yang et al., 2012). *OsCYP86A1* and *OsCYP86B1* were selected through the neighbor phylogram of protein sequences from *AtCYP86A1* and *AtCYP86B1* (Waßmann, 2015). *OsGPAT16*, which is phylogenetically closest to *AtGPAT5*, and *OsGPAT5*, which were expressed in the roots and significantly increased in salt stress, were selected among GPATs of rice (Safder et al., 2021). ATP-binding cassette, subfamily G (ABCG), known as suberin transporter, is co-expressed with suberin biosynthesis genes and is related to root suberization (Yadav et al., 2014). *OsABCG2* was selected with reference to its use in suberin-related gene expression analysis in rice (Singh et al., 2021). The suppression of AtESB1 expression was confirmed to increase root suberization (Wang et al., 2019). *OsESB1* was predicted to be putatively orthologous in rice from the model plants Arabidopsis thaliana using bioinformatics tools (Kreszies et al., 2018).

Similar to ABA-related gene expression results (**Figure 3**), suberization-related genes were upregulated at −1.0 and −1.5 MPa (**Figure 5**). To date, ABA-mediated suberization was broadly demonstrated in many plants, ABA-treated roots of Arabidopsis (Wang et al., 2020), and rice (Shiono et al., 2022) revealed huge accumulation of suberin lamella, which implies the close cooperation between ABA and suberization metabolisms. Hence, the current results are clearly in line with previous observations and provide evidence that the limited water supply leads to the regulatory network responses from ABA-signaling transduction to suberization via the modification in fatty acid composition. This provided evidence that the stress-tolerance mechanism of plants is activated under −1.0 and −1.5 MPa, not in −3.1 MPa. Therefore, our results imply that ABA-dependent metabolism in rice roots under drought condition is likely to be closely connected with suberization by promoting suberin monomer production (Kim et al., 2022).

### 4.4 Aquaporins activity in the root under drought condition

There is a total of 33 aquaporins in rice, which are classified into four types (Sakurai et al., 2008). Among the four types, plasma membrane intrinsic proteins (PIPs) and tonoplast instrinsic proteins (TIPs) exist in the plasma membrane and vacuole membrane, respectively. The PIP2 isoforms, regulating water permeability by both symplastic and apoplastic routes (Kaldenhoff and Fischer, 2006), are closely involved in water deficit. The transgenic rice overexpressing *OsPIP2;3*, localized in root endodermis, improved an adaptability against drought condition (Sun et al., 2021). OsPIP2;5 is predominantly localized in root endodermis and metaxylem and promotes water transport from endodermis to central xylem (Sakurai-Ishikawa et al., 2011). Our results showed a decreasing trend between different water potentials, and this finding is assumed that water movement toward the shoot could be strongly restricted to maintain water potential in root tissue under drought condition. OsPIP2;7, located in the endodermis and epidermis of root (Li et al., 2008), is highly promoted in response to ABA in drought-treated roots (Guo et al., 2006), and it is in an agreement with our observation. Accordingly, PIPs isoforms are likely to play different roles to regulate the inward/outward water flux in rice roots. TIPs isoforms, which regulate vacuolar water potential, are essential to control cell distention. Nguyen et al. (Nguyen et al., 2013) demonstrated that TIPs isoforms in rice plant were upregulated by water deficiency. Based on the current result, it is suggested that increased TIPs expression precisely regulate the water potential between both compartments, vacuole and cytosol.

### 4.5 Regulatory network among suberization, fatty acids synthesis, and aquaporins under drought condition

All key words (ABA, suberization, AQPs, and fatty acid) gather in the topic of water transport in cell membrane under drought stress in the roots. Through the previous discussion, a severe drought level may suggest that plants no longer develop tolerance mechanisms and die. Therefore, we focused on the −1.0 and −1.5 MPa results to suggest a hypothesis about the mechanism of water movement in cell membrane in drought. It has been demonstrated that the content of unsaturated fatty acids constituting the cell membrane increase in drought, creating spaces between lipids, which increases the fluidity of the membrane and ultimately activates the function of aquaporins (Martínez-Ballesta and Carvajal, 2016). However, the interaction of membrane constituent has not been fully known. As confirmed in the previous discussion, a stress tolerance mechanism occurred in −1.0 and −1.5 MPa. Taken together, this study suggests that ABA is involved as a signaling molecule in the interaction of aquaporins and suberin, and suberin lamellae formed on the membrane may interact with fatty acids and aquaporins to affect the fluidity and water transport. In **Figure 7**, it can be seen that the genes belonging to each keyword are highly correlated. *OsPIP2;5* was the variable accounting for 28.8% of PC2. Clearly, *OsAAO3* and *OsABA3* are negatively correlated with *OsESB1* in PC2. The defect of *atesb1* is compensated by ABA biosynthesis (Wang et al., 2019). The results are also supported with that plants use ABA to mitigate the adverse effects of suberization developed through suppression of *OsESB1* expression.

Further studies will be required to elucidate the interaction of each pathway in more detail to ultimately enhance drought tolerance mechanism. Since this study focused only on seedlings and roots, it will be needed to study the changes in each mechanism and the effect on other organs such as to leaves and grains.

## 5 Conclusions

Drought-resistant mechanism in rice roots is sophisticatedly regulated until permanent wilting point (−1.5 MPa) with an independent and/or concurrent process of ABA metabolism, suberization and AQPs activity, and thus rice roots are likely to facilitate water retention in cells by abundant suberin lamellae deposition as well as passive water absorption via activated AQPs and aerenchyma development. Based on some interesting findings and hypothesis, the current work is being focused on elucidating the regulatory networks between water-associated mechanisms under limited water environments.

## Data availability statement

The original contributions presented in the study are included in the article/**Supplementary Files**, further inquiries can be directed to the corresponding author/s.

## Author contributions

Conceptualization, G-EK and JS; validation, JS; investigation, G-EK; resources, G-EK and JS; writing-original draft preparation, G-EK; writing-review and editing, JS; supervision, JS; project administration; JS. All authors contributed to the article and approved the submitted version.
