# Supplementary material for: ABA-dependent suberization and aquaporin activity in rice (Oryza sativa L.) root under different water potentials
Source: Front Plant Sci. 2023 Sep 7;14:1219610. doi: 10.3389/fpls.2023.1219610 (PMC10512726; doi:10.3389/fpls.2023.1219610)
Supplement: Supplementary file 1 [file Table_1.docx]

Supplementary Material

ABA-dependent suberization and aquaporin activity in rice (*Oryza sativa* L.) root under different water potentials)

Ga-Eun Kim^1^, Jwakyung Sung^1*^

*** Correspondence:** jksung73@chungbuk.ac.kr; Tel.: +82-43-261-2512

# Supplementary Figures and Tables

**S1. Table 1.** Primer sequences used for qRT-PCR

| Gene symbol | Gene ID | Forward (5’-3’) | Reverse (5’-3’) | Tm (℃) | Product size (bp) |
| --- | --- | --- | --- | --- | --- |
| *OsCYP86A1* | *Os01g0854800* | GGCTGATCCAGCGAAAGA | AGGCCGCAGATGTTATCG | 55 | 190 |
| *OsCYP86B1* | *Os10g0486100* | CCTACAAGTTCACCGCCTTC | CTTGCTCTTGTCCCTCTTGG | 58 | 209 |
| *OsGPAT5* | *Os01g0782500* | ATATGGCACCGATCCTTCTG | CCCCTTCAGTTCCTTCATCA | 57 | 176 |
| *OsGPAT16* | *Os05g0457800* | GTACGAGGTGACCTTCCTCA | CACGCGGTACTTGTCCTT | 59.6 | 154 |
| *OsABCG2* | *Os01g0615500* | CTACTTCTCCCTCGTCGTCT | CCAGTACCTGGGGATGTTC | 59.6 | 175 |
| *OsESB1* | *Os01g0155300* | ACGCTCCAGAAGCTCCTCTT | GAACTCGCCTTCCTTGAACA | 56 | 174 |
| *OsNCED3* | *Os03g0645900* | GTTCAAGCTCCAGGAGATGC | GTTCCAGAGGTGGAAGCAGA | 57 | 154 |
| *OsABA2* | *Os03g0810800* | AACTGCGTATCTCCCTATGC | TAGAGCACTGCTTGAGCAAC | 55 | 167 |
| *OsABA3* | *Os06g0670000* | AAAGGATGTGCCACTGAACC | TTGAAGCAGCTACGGTTCCT | 60 | 175 |
| *OsAAO3* | *Os07g0119400* | GTGACGAACGACAACATCAT | GAGGGGAAGTAGACGAAGGT | 60 | 200 |
| *OsPYL6* | *Os03g0297600* | AACTACCTCTCGGTCACCAC | GAGAGACTGGAGGTTGCACT | 58 | 159 |
| *OsSAPK10* | *Os03g0610900* | GACCTGAAGCTGGAGAACAC | GTTACTCCACACGACCACAC | 58 | 200 |
| *OsPIP2;3* | *Os04g0521100* | CGGTGTTCATGGTTCACTTG | CCTCAGGACGTACTGGTGGT | 60 | 182 |
| *OsPIP2;5* | *Os07g0448400* | GGCATGATCTTCATCCTGGT | TGTCGTAGAACGAGCTCTGG | 60 | 193 |
| *OsPIP2;7* | *Os09g0541000* | CCTTCATCCCCGTACTCGT | CACCCAGAAGATCCAGTGGT | 59 | 167 |
| *OsTIP1;2* | *Os01g0975900* | GAACATCAGCCTGGTGAAGG | TCATGACGATCTCGAACACC | 55 | 167 |
| *OsTIP3;1* | *Os10g0492600* | TACTACGCGACGGTGATCG | GAGCCAGTACACCCAGTGGT | 59 | 189 |
| *OsTIP3;2* | *Os04g0527900* | CACCACTGGGTTTACTGG | ACGGGAAGCCGAGTATCT | 50 | 119 |
| *OsTIP4;1* | *Os05g0231700* | ATCGCTGGTGGCAACTTC | GAGGCTCATGGGTCCTCTT | 59 | 178 |
| *Actin* | | TGTATGCCAGTGGTCGTACC | CCAGCAAGGTCGAGACGAA | 57 | 186 |
